# Combined effects of cigarette smoking, alcohol drinking and eNOS Glu298Asp polymorphism on blood pressure in Chinese male hypertensive subjects

**DOI:** 10.18332/tid/110678

**Published:** 2019-08-02

**Authors:** Zhe Hong, Liying Pan, Zhangqing Ma, Yue Zhu, Zongyuan Hong

**Affiliations:** 1 Tongji Hospital of Tongji University School of Medicine, Shanghai, China; 2 The First Affiliated Hospital of Wannan Medical College, Wuhu, China; 3 Laboratory of Quantitative Pharmacology, Wannan Medical College, Wuhu, China

**Keywords:** cigarette smoking, alcohol drinking, Glu298Asp polymorphism, combined effect, blood pressure

## Abstract

**INTRODUCTION:**

Genetic factors and lifestyle exposures, as well as their combinations, play important roles in the development of hypertension. We examined whether cigarette smoking, alcohol drinking and the Glu298Asp polymorphism of the endothelial nitric oxide synthase (eNOS) gene generate combined effects on blood pressure (BP) in hypertensive subjects.

**METHODS:**

A total of 342 essential hypertensive subjects were recruited from Susong community in Anhui province, China, from July 2017 to January 2018, and the plasma biochemical parameters and the genotype on Glu298Asp polymorphism were determined.

**RESULTS:**

There were no gender differences in the distributions of alleles and genotypes in hypertensive subjects. The proportions of cigarette smoking and alcohol drinking in male hypertensive subjects were remarkably higher than those in the females (p<0.001). The systolic blood pressure (SBP) and diastolic blood pressure (DBP) levels of mutant genotypes (*Glu/Asp* and *Asp/Asp*) were significantly higher than those of wild genotype (*Glu/Glu*) (p=0.013 and 0.026, respectively) in male hypertensive subjects. Moreover, the SBP and DBP levels of the mutant genotype were remarkably higher than those of wild genotype in both cigarette smoking and alcohol drinking male hypertensive subjects (p=0.034 and 0.043, respectively).

**CONCLUSIONS:**

Cigarette smoking, alcohol drinking and the Glu298Asp polymorphism of the eNOS gene generate combined effects that increase the susceptibility of the mutant genotype to BP in Chinese male hypertensive subjects.

## INTRODUCTION

Human hypertension is one of the most serious risk factors for cardiovascular disease^1,2^. One-fifth of the adult population suffers from the disease, and 95% of the cases are ‘essential hypertension’ with uncertain aetiology^3^, and thus the term essential hypertension (EH) is employed to describe such situations^4^. EH is considered a multi-factorial disease resulting from the interplay of genetic, lifestyle, and environmental factors^5,6^. It is estimated that 30–40% of the variation in blood pressure could be attributed to heritability^7^. The genetic determinants interact with established environmental determinants of blood pressure levels including age, sex, tobacco use, sodium intake, alcohol consumption etc. to produce the final disease phenotype^8,9^.

Biological nitric oxide (NO) is mostly produced enzymatically from L-arginine by a family of three NO synthases (NOSs), neuronal NOS (nNOS or NOS1), inducible NOS (iNOS or NOS2) and endothelial NOS (eNOS or NOS3)^10^. The eNOS, which is mainly produced in vascular endothelial cells, is a vascular smooth muscle relaxing factor that plays an important role in the regulation of BP and regional blood flow. Administration of L-arginine decreases BP, while the administration of NOS inhibitor increases BP^11^. Endothelium-dependent blood vessel relaxation is impaired in hypertensive patients and in animal models of hypertension induced by hereditary trait, or Goldblatt’s clamp. Impaired relaxation improves as hypertension normalizes^12^. Furthermore, an increase in BP was observed in mice deficient in the eNOS gene^13^. In contrast, eNOS gene delivery was reported to reduce systemic BP^14^. These findings indicate that the eNOS gene is a candidate gene for human hypertension. The human eNOS gene is located at 7q35-36 and spans 21 kb with 26 exons and 25 introns^15^, and has been shown to be polymorphic. It is reported that the Glu298/Asp (G894→T conversion at nucleotide position 894) polymorphism of the eNOS gene decreases NO production and subsequently influences the development of EH and atherosclerosis^16,17^.

Smoking and alcohol drinking habits, either in social settings or at home, are common among Chinese, especially men, and the effects of cigarette smoking and alcohol consumption on cardiovascular disease are very diverse. It has been documented that cigarette smoking may produce an acute rise in BP and be a crucial risk factor for coronary spasm^18^. Moreover, smokers have reduced endothelial-dependent vessel relaxation, accelerated atherosclerosis plaque development and premature coronary artery diseases. Cigarette smoking is also a rich source of exogenous NO that can affect endogenous NO production^19^. On the other hand, chronic consumption of ethanol is known to produce several adverse cardiovascular effects, including hypertension^20^. Further, several studies have reported an association between alcohol consumption and high BP^21,22^, and the prevalence of systolic hypertension is greater in moderate and heavy drinkers than in non-drinkers^23^.

The above knowledge naturally leads to the hypothesis of whether smoking, alcohol drinking and eNOS Glu298Asp polymorphism generate combined effects on BP in hypertensive subjects. To examine this hypothesis, we recruited 342 EH patients with comprehensive phenotypic data from Susong community in Anhui province, China, and their blood samples were collected to determine the genotype on Glu298Asp polymorphism and plasma biochemical parameters for investigating the combined effects of smoking, alcohol drinking and the polymorphism on BP.

## METHODS

### Study subjects

A total of 342 hypertensive patients (178 males, 164 females), who were from Susong community in Anhui province, China, from July 2017 to January 2018, were recruited into the study. These patients met the following criteria: 1) Systolic blood pressure (SBP) in the range of 140–200 mmHg, diastolic blood pressure (DBP) 90–120 mmHg, or both, on 3 separate occasions; 2) Age between 21–65 years and a body mass index (BMI) <33 kg/m^2^; 3) Patients receiving antihypertensive drugs treatment or taken other drugs in two weeks before the study, showing biological signs of malignant disease and with secondary hypertension, were excluded. 4) Not having cerebrovascular, liver, diabetes mellitus, hyperlipidaemia, coronary heart diseases and severe abnormalities of echocardiography; and 5) Not being nursing or pregnant women and not having experienced stenosis of bi-renal artery or an operation on duodenum. This study and related protocols were approved by the Medical Ethics Committee of Wannan Medical College. Written informed consent was obtained from all the subjects recruited.

### Measurement of BP, body weight and height

After the patients rested supinely for about one hour and no alcohol, cigarette, coffee or tea etc. was consumed, the SBP and DBP were measured on the right arm of these patients in supine position three times with one minute intervals by a mercury sphygmomanometer with an appropriately sized cuff by trained nurses. If the difference among the three measurements was more than 4 mmHg, the patient was asked to rest for 5 minutes and the blood pressure was measured again. The SBP and DBP were expressed as the mean of the three measurements. The patients’ body weight and height were also measured.

### Measurement of plasma biochemical parameters

Ten millilitres venous blood from each patient at fasting was drawn into a vacutainer (Becton Dickinson, Franklin Lakes, NJ) containing EDTA and citrate for DNA and plasma biochemical parameters, respectively. The vacutainers were then kept on ice and subsequently centrifuged for 10 min at 4000×g at 4°C and stored at -80°C. The plasma levels of total cholesterol, triglyceride, high-density lipoprotein, low-density lipoprotein etc. were then detected using a BECKMAN CX5CE clinical analyser system (Beckman Coulter, Inc., Brea, CA).

### Extraction of DNA and genotyping of polymorphisms

The genomic DNA was extracted according to the instructions of the GoldMag DNA Purification Kit. The eNOS Glu298Asp (G894→T) polymorphism was genotyped by PCR amplification of genomic DNA and subsequent restriction-endonuclease BanII (New England Biolabs, Beverly, MA) digestion. The PCR fragment size was 249 base pairs (bp). When the G of the G894T variant was present, a 249bp fragment was reduced to 165bp and 84bp fragments, whereas the 249bp fragment was not digested if T was present.

### Statistical analysis

All data were analysed by the SAS 6.12 software package. Continuous variables were expressed as mean ± standard deviation (SD), and categorical variables were expressed as percentages. The differences between men and women were calculated using the unpaired Student’s t-test for continuous data and the chi-squared (χ^2^) test for categorical data. The differences in the distributions of genotypes and allele frequencies between male and female hypertensive subjects were evaluated by logistic regression analysis. The relationship between genotypes and BP levels was performed by multiple linear regression analysis. Considering that demographic factors may affect BP levels, the patient’s age, BMI, cigarette smoking and alcohol drinking status etc. were adjusted in the regression model. In order to investigate the combined effects of cigarette smoking, alcohol drinking and eNOS Glu298Asp polymorphism on BP, analysis of covariance (ANCOVA) was used to test the difference in SBP or DBP among different genotypes, and comparison between groups was assessed by the Student-Newman-Keuls multiple range test (SNK, q-test). A p<0.05 was considered to be statistically significant.

## RESULTS

### Demographic and clinical characteristics of study subjects

The demographic and clinical characteristics of the 178 male and 164 female hypertensive subjects are summarized in Table 1. No differences between the two groups were noted with respect to age and plasma biochemical parameters (total triglyceride, total cholesterol, high-density lipoprotein and low-density lipoprotein). The mean height and weight of the males were significantly higher than those of the females (p<0.001), whereas the BMI for females was greater than that for males (p=0.038). These were consistent with male and female physiological characteristics. Interestingly, the proportions of cigarette smoking and alcohol drinking in the males (44.4% and 42.1%, respectively) were remarkably higher than those in the females (6.1% and 3.0%, respectively) (p<0.001).

**Table 1:** Demographic and clinical characteristics of study subjects

*Variable*	*Male (n=178 )*	*Female (n=164 )*	*p*
Age (years)	48.5 ± 7.9	48.4 ± 7.1	0.901
Height (cm)	165.7 ± 6.3	156.0 ± 5.4	<0.001
Weight (kg)	65.8 ± 10.8	55.3 ± 10.2	<0.001
BMI (kg/m^2^)	23.9 ± 3.3	24.7 ± 3.8	0.038
SBP (mmHg)	156.0 ± 18.8	159.7 ± 16.0	0.052
DBP (mmHg)	100.2 ± 8.2	98.5 ± 6.4	0.033
TG (mmol/L)	1.2 ± 0.7	1.1 ± 0.8	0.217
CHOL (mmol/L)	4.8 ± 0.9	4.9 ± 0.8	0.280
HDL (mmol/L)	1.3 ± 0.3	1.3 ± 0.3	1.000
LDL (mmol/L)	2.9 ± 0.8	3.0 ± 0.7	0.220
Smoking, yes/no (%)	79/99 (44.4)	10/154 (6.1)	<0.001
Alcohol drinking, yes/no (%)	75/103 (42.1)	5/159 (3.0)	<0.001

BMI: body mass index, SBP: systolic blood pressure, DBP: diastolic blood pressure, TG: total triglyceride, CHOL: total cholesterol, HDL: high-density lipoprotein, LDL: low-density lipoprotein. Categorical variables are presented as number (%), and continuous variables are summarized as mean ± SD.

In addition, the DBP level in the males was markedly higher than that in the females (p=0.033), while the difference in SBP level between the two groups was not significant.

### Analysis of genotype and allele frequencies of Glu298Asp polymorphism

The prevalence of the eNOS alleles in the males and females satisfied the Hardy-Weinberg equilibrium law. The genotype frequency in codon 298 of the eNOS gene in the males was 77.0% for *Glu/Glu* and 23.0% for *Glu/Asp*+*Asp/Asp*, and allele distributions of Glu and Asp were 87.1% and 12.9%, respectively (Table 2). In the females, the genotype frequency was 73.8% for *Glu/Glu* and 26.2% for *Glu/Asp*+*Asp/Asp*, and allele frequencies of Glu and Asp were 86.3% and 13.7%, respectively. There were no significant differences in genotype and allele frequencies between the males and females. The odds ratios were 1.19 (95% CI: 0.69–1.68) and 1.07 (95% CI: 0.63–1.51), respectively, and the differences were also not significant (Table 2).

**Table 2:** Distributions of genotype and allele frequency of eNOS Glu298Asp polymorphism in male and female hypertensive subjects by logistic regression analysis

*Genotype and Allele*	*Male (n=178 ) n (%)*	*Female (n=164 ) n (%)*	*OR ( 95% CI)*	*p*
**Genotype**				
*Glu/Glu* (Reference)	137 (77.0)	121 (73.8)	1.0	
*Glu/Asp*+*Asp/Asp*	41 (23.0)	43 (26.2)	1.19 (0.69–1.68)	0.494
**Allele frequency**				
Glu (Reference)	310 (87.1)	283 (86.3)	1.0	
Asp	46 (12.9)	45 (13.7)	1.07 (0.63–1.51)	0.759

eNOS: endothelial nitric oxide synthase, OR: odds ratio, CI: confidence interval. Categorical variables are presented as number (%).

### Combined effects of cigarette smoking, alcohol drinking and Glu298Asp polymorphism on BP

A linear regression analysis, which was adjusted for confounding factors such as age, BMI, cigarette smoking and alcohol drinking etc., showed that the mutant genotype (*Glu/Asp* and *Asp/Asp*) individuals had higher SBP and DBP levels than *Glu/Glu* genotype in the males (p=0.013 and 0.026, respectively, Table 3). In order to investigate the effect of cigarette smoking and alcohol drinking on BP levels of the wild genotype and the mutant genotype subjects, we analysed, by ANCOVA, the interaction between cigarette smoking as well as alcohol drinking and eNOS *Glu/Asp*. The results showed that the SBP and DBP levels of the mutant genotype were markedly higher than those of wild genotype in cigarette smoking or alcohol drinking male hypertensive subjects (p=0.034 or 0.043, respectively). The DBP level of the mutant genotype was higher in cigarette smoking or alcohol drinking male hypertensive subjects than in the non-smoking or non-drinking (p=0.041), although the difference was not found in the SBP (Table 4). The same differences were not found in total subjects (data not shown). These findings indicate that the mutant genotype individual’s BP is more easily influenced by cigarette and alcohol in male hypertensive patients.

**Table 3:** The relationship of eNOS Glu298Asp polymorphism and blood pressure^a^ in hypertensive subjects by linear regression analysis

*Genotype*	*n*	*Mean ± SD*	*Coefficient*	*SE*	*p*
**Male**					
SBP (mmHg)					
*Glu/Glu*	137	154.3 ± 18.1	0		
*Glu/Asp+Asp/Asp*	41	161.8 ± 20.2	7.92	3.19	0.013
DBP (mmHg)					
*Glu/Glu*	137	99.5 ± 7.8	0		
*Glu/Asp+Asp/Asp*	41	102.8 ± 9.6	3.11	1.58	0.026
**Female**					
SBP (mmHg)					
*Glu/Glu*	121	159.7 ± 16.2	0		
*Glu/Asp+Asp/Asp*	43	159.8 ± 15.8	-0.94	2.57	0.716
DBP (mmHg)					
*Glu/Glu*	121	98.7 ± 6.3	0		
*Glu/Asp+Asp/Asp*	43	99.4 ± 6.9	0.99	1.19	0.406

eNOS: endothelial nitric oxide synthase, SBP: systolic blood pressure, DBP: diastolic blood pressure, SD: standard deviation, SE: standard error. Continuous variables are summarized as mean ± SD.

a Adjusted for age, body mass index, smoking status, and alcohol drinking.

**Table 4:** Combined effects of smoking, drinking and genotype on blood pressure^a^ in male hypertensive subjects by SNK (following ANCOVA)

*Smoking (+/-) or Drinking (+/-)*	*G*	*Genotype*	*n*	*Mean ± SE*	*p*		
					*Vs G4*	*Vs G3*	*Vs G2*
**SBP (mmHg)**							
(-) and (-)	1	*Glu/Glu*	95	153.4 ± 1.8	0.031	0.850	0.023
	2	*Glu/Asp+Asp/Asp*	27	161.6 ± 3.3	0.450	0.057		
(+) and (+)	3	*Glu/Glu*	42	153.9 ± 2.3	0.034		
	4	*Glu/Asp+Asp/Asp*	14	164.3 ± 3.6			
**DBP (mmHg)**							
(-) and (-)	1	*Glu/Glu*	95	99.8 ± 0.9	0.039	0.892	0.771
	2	*Glu/Asp+Asp/Asp*	27	100.2 ± 1.1	0.041	0.703	
(+) and (+)	3	*Glu/Glu*	42	99.6 ± 1.3	0.043		
	4	*Glu/Asp+Asp/Asp*	14	105.1 ± 2.2			

ANCOVA: analysis of covariance, SBP: systolic blood pressure, DBP: diastolic blood pressure, SE: standard error, SNK: Student-Newman-Keuls, G: group. a Adjusted for age, gender, and body mass index.

## DISCUSSION

The human essential hypertension is a multi-factorial disease. Its cause and development are involved in genetic factors, environmental factors and lifestyle exposures, as well as their interactions. In the present study, we used the candidate gene approach to explore whether cigarette smoking, alcohol drinking and the gene encoding endothelial nitric oxide synthase, which is a key enzyme producing NO, generated effects on BP in hypertensive subjects. We demonstrated that there was a significant combined effect of smoking, drinking and the Glu298Asp polymorphism of the eNOS gene on BP in Chinese male hypertensive subjects. This is the first report of the combined effects of these three factors on BP.

NO, a vascular smooth muscle relaxing factor, which is mainly produced by eNOS in vascular endothelial cells, plays an important role in the relaxation of vessels, inhibition of the vascular smooth muscle cell proliferation, and BP regulation. A study showed that a reduction in basal NO release might predispose individuals to hypertension, thrombosis, vasospasm and atherosclerosis; and restoration of NO activity could induce regression of pre-existing intimal lesions^19^. Whereas the formation of NO is controlled by eNOS, eNOS activity and/or production can directly influence the regulation of BP^11-14^. The Glu298Asp polymorphism is a nucleotide substitution at the open reading frame on the eNOS gene that causes amino acid substitution of glutamic acid to aspartic acid at a codon in the 298th position^24^. Though there is no evidence that the acid substitution changes eNOS activity, computer analysis has revealed that the Glu298Asp mutation induces a conformational change from helix to tight turning of the eNOS^25^, which indicates that homozygosity for the Asp298 could result in a reduction in eNOS activity. Philip et al.^26^ reported enhanced vasoconstriction in response to phenylephrine in Asp298 homozygotes that may be ascribed to an impaired endothelial NO modulation of adrenergic vasoconstriction. Moreover, published bovine^27^ and human^28^ heme domain eNOS structures revealed that Glu298 (Glu300 in bovine) was part of the catalytic heme domain. Hence, it is conceivable that this site may be part of a yet-to-be-identified protein-protein interaction site^29^ that is sensitive to Glu→Asp substitution. These findings show that the amino acid substitution (Glu298→Asp) of the eNOS gene might influence the eNOS activity and boost cardiovascular events, such as hypertension. Our results also suggest that hypertensive subjects bearing mutant genotypes (*Glu/Asp* and *Asp/Asp*) have higher BP than those with wild genotype (*Glu/Glu*) in Chinese male hypertensive subjects. This result is consistent with previous studies^16,17^.

However, interestingly, the trend of BP elevation only appeared in male hypertensive patients and was not observed in the females of our study. This phenomenon could not be completely explained by Glu298Asp polymorphism because in our study there was no difference in the distributions of alleles and genotypes between male and female patients. At least, it may in part be explained with smoking and/or alcohol consumption, because in our study subjects the proportions of cigarette smoking and alcohol drinking in male hypertensive subjects were remarkably higher than in the females. As mentioned above, hypertension is a multi-factorial disease. Besides genetic factors, smoking and alcohol consumption lifestyle exposures as well as their interactions, such as gene-gene, gene-smoking, gene-drinking etc., also affect the cause and development of hypertension. In fact, cigarette smoking or alcohol drinking could per se elevate BP^9,18,20,23,30^, while smoking-gene polymorphisms might also generate combined effects on BP by affecting the endogenous NO production or eNOS activity^19^. It has been reported that smoking extracts contain oxygen free radicals, which inactivate NO or directly damage endothelial cells^31,32^. Wang et al.^19^ reported that associations between eNOS polymorphism and protein levels and enzyme activities were modifiable by smoking, and the effects of smoking were dependent on the eNOS genotypes. They further indicated that the interaction of smoking and Glu298Asp (G894→T) polymorphism could reduce the eNOS protein levels and/or the enzyme activities. In addition, a positive association between alcohol drinking and both SBP and DBP was well established^23,30,33^ by raising blood cortisol levels and increasing the excretion of urinary catecholamine and their metabolites, which could possibly lead to elevated BP^34^. Our results indicate that in male hypertensive patients with both cigarette smoking and alcohol drinking, the SBP and DBP levels of the mutant genotype were higher than those of the wild type. This implies that the mutant genotype is more easily affected by smoking and/or drinking and the interactions between smoking-drinking, smoking-gene as well as drinking-gene compared with the wild genotype in male hypertensive subjects.

### Strengths and limitations

We found, for the first time, that smoking, drinking and the Glu298Asp polymorphism of the eNOS gene may generate a significant combined effect on BP in male hypertensive subjects, which would provide a novel approach to prevent human hypertension. One limitation of our study was the relatively small sample size that resulted in limited power for detecting significant associations. Another limitation was that we did not measure eNOS expression or NO levels in plasma etc. biochemical indicators in the enrolled hypertensive patients. Thus, we do not have direct evidence to evaluate the effect of the eNOS Glu298Asp polymorphisms on BP, although previous studies support this hypothesis. It is also a limitation that we did not set up a control group with normotensive subjects to examine if the mutant genotype is present in higher frequency among those with hypertension and compare the frequency among smokers and alcohol drinkers among the controls.

## CONCLUSIONS

Cigarette smoking, alcohol drinking and Glu298Asp polymorphism may generate combined effects that increase the susceptibility of the mutant genotype to BP in Chinese male hypertensive subjects.
